# Effectiveness and Cost-benefit Evaluation of a Comprehensive Workers’ Health Surveillance Program for Sustainable Employability of Meat Processing Workers

**DOI:** 10.1007/s10926-017-9699-9

**Published:** 2017-03-24

**Authors:** Berry J. van Holland, Michiel F. Reneman, Remko Soer, Sandra Brouwer, Michiel R. de Boer

**Affiliations:** 1 Department of Health Sciences, Community and Occupational Medicine, University Medical Center Groningen, University of Groningen, Groningen, The Netherlands; 2 Department of Rehabilitation Medicine, Center for Rehabilitation, University Medical Center Groningen,, University of Groningen, Groningen, The Netherlands; 3 Expertise Center of Health, Social Care and Technology, Saxion University of Applied Sciences, Enschede, The Netherlands; 4 University Medical Center Groningen, Groningen Spine Center, University of Groningen, Groningen, The Netherlands; 5 Department of Health Sciences, Faculty of Earth and Life Sciences, Institute for Health Sciences, VU University, Amsterdam, The Netherlands

**Keywords:** Meat-packing industry, Workplace health promotion, Intervention study, Stepped wedge trial, Return on investment

## Abstract

*Objective* To evaluate the effectiveness of a comprehensive workers’ health surveillance (WHS) program on aspects of sustainable employability and cost-benefit. *Methods* A cluster randomized stepped wedge trial was performed in a Dutch meat processing company from february 2012 until march 2015. In total 305 workers participated in the trial. Outcomes were retrieved during a WHS program, by multiple questionnaires, and from company registries. Primary outcomes were sickness absence, work ability, and productivity. Secondary outcomes were health, vitality, and psychosocial workload. Data were analyzed with linear and logistic multilevel models. Cost-benefit analyses from the employer’s perspective were performed as well. *Results* Primary outcomes sickness absence (OR = 1.40), work ability (B = −0.63) and productivity (OR = 0.71) were better in the control condition. Secondary outcomes did not or minimally differ between conditions. Of the 12 secondary outcomes, the only outcome that scored better in the experimental condition was meaning of work (B = 0.18). Controlling for confounders did not or minimally change the results. However, our stepped wedge design did not enable adjustment for confounding in the last two periods of the trial. The WHS program resulted in higher costs for the employer on the short and middle term. *Conclusions* Primary outcomes did not improve after program implementation and secondary outcomes remained equal after implementation. The program was not cost-beneficial after 1–3 year follow-up. Main limitation that may have contributed to absence of positive effects may be program failure, because interventions were not deployed as intended.

## Introduction

Working in the meat processing industry consists of repetitive, monotonous and physically demanding tasks [1]. Workers are exposed to several occupational health hazards simultaneously [2]. Most common occupational injuries and illnesses reported are musculoskeletal disorders (MSDs), skin disorders, hearing disorders and infectious diseases. In general, it is known that these disorders and diseases increase the chance for sickness absence and reduced work ability [3]. If disorders or diseases are severe enough, they may lead to early retirement or disability pension [4] and thus have considerable economic consequences [5].

On top of the health hazards come societal developments such as an aging workforce and rising retirement age [6]. Higher age is an indicator for lower work ability [7]. This calls for intervention programs aimed at sustainable employability by reducing sickness absence and improving/maintaining work ability. Sustainable employability is defined as the opportunity to perform work with preservation of health and wellbeing during one’s working life, now and in the future [8]. Considering the characteristics of the work in the meat processing industry, occupational health hazards, and societal developments it is of great importance to address the sustainable employability of workers in this industry.

In a recent systematic review limited evidence was found for favorable effects of interventions aimed at sustainable employability in ageing workers [9]. In the meat processing industry several health interventions have been deployed. Strong evidence was presented for 100% effectiveness of Q fever vaccination, moderate evidence for skin protection, and low level evidence for ergonomics programs [10]. However, no interventions aimed at sustainable employability were identified. Employers and employees are more aware now of their responsibility towards sustainable employability and have created more interest in interventions such as health promotion programs [11]. In particular, job-specific health surveillance seems to be promising, as was demonstrated in another occupation (nursing) [12]. Since meat processing workers are a vulnerable population at risk for reduced employability, they may benefit from a job-specific program aimed at sustainable employability.

The present study evaluated the POSE program (Promotion of Sustained Employability), which is a comprehensive workers’ health surveillance (WHS) program developed by commercial parties in collaboration with an occupational health service [13]. The program consists of a WHS program combining elements from occupational medicine (e.g., health surveillance, and interventions aimed at a healthy lifestyle [14]) and rehabilitation medicine (e.g., functional capacity evaluation (FCE) [15], and interventions aimed to improve physical capacity). The POSE program offers workers a custom-made risk profile and, if necessary, an intervention plan using an integral approach with the aim of offering them the opportunity to increase their probability of sustained employability and reduce potential health risks. The objectives of this study were to investigate the effectiveness and cost-benefit of the POSE program compared to care as usual (CAU) in a randomized stepped wedge trial with 1–3-year follow-up. The following research questions were addressed:

1. What is the effect of the POSE program compared to CAU on the primary outcome measures work ability, sickness absence, and productivity?
2. What is the effect of the POSE program compared to CAU on the secondary outcome measures psychosocial workload, subjective health status, and vitality?
3. Which effect does the POSE program have on costs and benefits from the employer’s perspective?

A pilot study was conducted before the onset of this stepped wedge trial in which the POSE program led to improved work ability (unpublished material). This result was used for the power calculation of the present trial [13]. Based on the findings in the pilot study we hypothesized that the POSE program would be effective on primary and secondary outcomes and as a consequence be cost-beneficial.

## Methods

The CONSORT statement was used to describe this study [16]. The Medical Ethical Committee of the University Medical Center Groningen (the Netherlands) declared that the Medical Research involving Human Subjects Act did not apply to the current study.

### Trial Design and Study Participants

#### Design

**Fig. 1:**
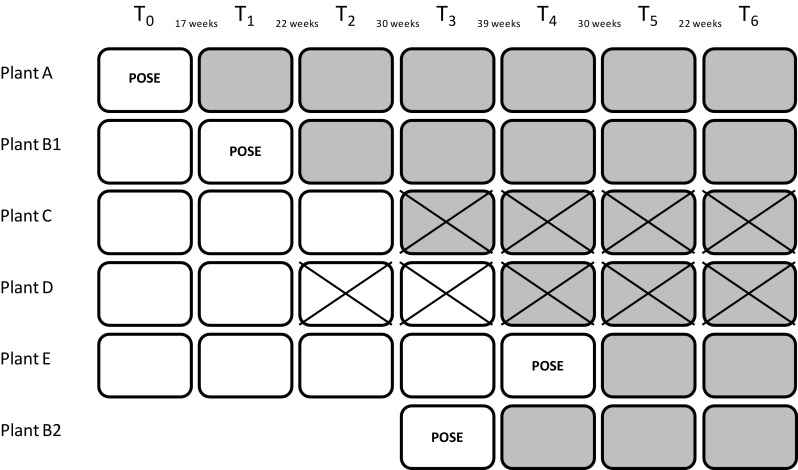
Final trial design. *White boxes* refer to periods in the control condition, *grey boxes* refer to periods in the intervention condition. At the POSE sign, the program was implemented. *POSE* Promotion of sustained employability

The study was designed as a stepped wedge trial with follow-up measurements within a 1–3-year period after start of the intervention. A detailed description is published in the design paper [13]. In brief, the study was carried out within a large Dutch meat processing company from february 2012 to march 2015. At the start of the study, 15 company plants were available of which five fulfilled the inclusion criteria (sufficient number of workers required for sufficient power, budget for the POSE program). The order of program implementation of those five plants was randomly assigned. During the course of the study, reorganizations within the company forced the researchers to change the study design. The final design is displayed in Fig. 1. The closure of two of the five plants (C and D) made it necessary to include another group of participants in the study. They were recruited from an already participating plant (B1), and introduced as plant B2.

#### Participants

All contracted workers (n = 986) were invited to participate in the study. Workers were eligible if they were contracted personnel, performed paid labor for at least 12 h per week at the company (as defined by Statistics Netherlands), agreed to participate in the POSE program, and provided informed consent to participate in the study.

### Workers’ Health Surveillance

The POSE program has previously been described in detail [13]. The program was designed to identify workers who were at risk for reduced employability. Various screening tests were administered, in order to create the risk profile. For this purpose several constructs were addressed such as physical and mental health, and physical and mental work capacity. An online questionnaire focused on work ability, health, and lifestyle. Physical measurements were performed at the workplace and focused on biometric components (e.g., blood pressure, cholesterol, vision, hearing) and functional capacity (FCE addressing material handling, postural tolerance, coordination and repetition, hand and finger strength, and energetic capacity) [17, 18]. A registered vocational physiotherapist conducted a counseling session with the participant and discussed the results of the screening tests. Based on the outcomes of the screening tests, participants received recommendations on future interventions, such as a visit to the general practitioner, physiotherapist, or dietician.

### Care as Usual

Regular (occupational) healthcare, unrelated to the POSE program, was considered CAU. Programs already running within the company were considered CAU as well. These could be company fitness programs, healthy canteens, etc.

### Outcomes

#### Effectiveness Evaluation

Primary outcomes: sickness absence, work ability, productivity. Sickness absence data were provided by the company. Sickness absence days (according to calendar days) were calculated for 1 year prior to baseline and for the periods in between measurement points. Partial sickness absence was taken into account by multiplying absence percentage and number of corresponding absence days. Absence periods of more than 2 days were recalculated by multiplying by 5/7, to ensure that weekends were excluded. Next, data were recalculated to days per year. Finally, absence days were dichotomized. The cut-off was set at nine work days per year, as this matches the average yearly absence rate at the company (approx. 4.5%). Work ability was measured with the work ability index (WAI) [19] for which a sum score was calculated, ranging from 7 to 49. The WAI was once electronically collected during the POSE program assessments and at the other time points by paper questionnaire. For calculation of the disease categories we used the number of diseases according to one’s own opinion. When such items were missing we used diagnosed diseases, if possible. Otherwise, it was assumed that there were no diseases. The WAI score was only calculated if there were no missing data on main items. Test–retest reliability of the WAI is acceptable [20]. Productivity at the individual level was measured by self-report using the quality and quantity (QQ) questionnaire [21]. The recall period for productivity was one work day. Only the quantity item of the questionnaire was used. The score was dichotomized into full productivity (10) and productivity loss (0–9) [22].

Secondary outcomes: psychosocial workload, health status, vitality. Aspects of the psychosocial workload were measured by a short form of the second version of the copenhagen psychosocial questionnaire (COPSOQ II) [23, 24]. These aspects were quantitative work demands, work pace, autonomy, possibilities for development, meaning of work, job satisfaction, social support from supervisor, social support from colleagues, and sense of community. For all subscales a score was calculated (0–8), except for job satisfaction (1 item; 0–3). Self-reported health status was evaluated by the Dutch version of the EuroQol-5D [25]. It consists of five short questions on various health domains and a health thermometer (0–100). The valuation of EQ-5D scores is based on the Dutch tariff, and results in scores between −0.329 and 1, with scores below zero meaning a worse quality of life than death, zero equal to death and one meaning perfect quality of life. Self-reported vitality was assessed by a subscale of the RAND-36 questionnaire. This scale consists of four questions on a five-point Likert scale from which a scale score was calculated (0–100). The RAND-36 is highly reliable and has satisfactory construct validity [26].

#### Covariates

Personal and work characteristics were assessed as covariate in this study. Most personal and work characteristics were retrieved from the company registry (e.g., age, gender, contract hours, job tenure) and POSE program (education).

#### Cost-Benefit Evaluation

The cost-benefit of the POSE program was evaluated from the employers’ perspective. The cost-benefit analysis evaluated the total costs of the POSE program for the company and compared absenteeism and presenteeism costs before and after the POSE program. Costs (in Euros) included:

Direct costs of the POSE program: the costs of the program itself (assessments and counseling).

Absenteeism costs. Average salaries per plant department were obtained from the company. The gross annual salary including holiday allowances and premiums was recalculated to salary per workable day (day salary = gross salary/236 days) [27]. The number of absence days per period was recalculated to absenteeism costs expressed in monetary terms using these salaries (number of absence days * day salary).

Presenteeism costs. Productivity loss at work was assessed using the QQ questionnaire [21], consisting of a quantity and a quality item, with both scores ranging from 0 (nothing) to 10 (regular productivity). Recalculation resulted in a productivity loss score between 0 and 1 [1 − (quality * quantity/100)]. The productivity loss score was multiplied by the number of work days (minus absence days) in a given period. The number of days lost were expressed in monetary terms using day salary (presenteeism days * day salary). Presenteeism per period was calculated by averaging the productivity loss scores from two measurement points because no continuous scores for a period were available. No average could be calculated for the period prior to baseline, and therefore the period before baseline was not included in the cost-benefit analysis.

### Sample Size

The sample size calculation was based on the effectiveness with regard to work ability and resulted in a sample size of 44–46 participants per plant assuming participation of four plants. The sample size calculation for this study has been described in the design paper [13].

### Data Analysis

#### Non-response Analysis

POSE program participants (control and experimental group) were compared to non-participants with regard to age, gender, job tenure, contract hours, and total sickness absence duration one year before baseline. Group differences were analyzed with Mann–Whitney U tests and Fisher’s exact test and considered significant if p < 0.05.

#### Effectiveness

Analyses on the effectiveness of the intervention were performed in SPSS version 22.0 for Windows (IBM Corp., Armonk, New York, USA). The effect of the trial on the primary and secondary outcomes was analyzed using linear (for the continuous outcomes) and logistic (for the binary outcomes) multilevel analyses including a random coefficient for the individual [28], and a fixed coefficient for time. The latter parameter was included to adjust for any possible time effects. Because individuals crossed over from the control condition to the experimental condition, they acted as their own controls in all analyses. Analyses were adjusted for baseline values of the outcome of interest and for age. All tests were performed two-sided, assuming an alpha of 0.05.

#### Sensitivity

To control whether long-term sickness absence affected the results of the primary analysis, multiple sensitivity analyses were performed. First, the analyses on sickness absence were restricted to participants without sickness absence periods of more than 200 standardized days/year (n = 17) or participants without sickness absence periods of more than 100 standardized days/year (n = 47). Second, sickness absence days due to exceptional causes (i.e. unrelated to the risks in the POSE program, such as cancer, and traffic or horse accidents; as determined by the occupational health service; total n = 16) were set to zero. Sickness absence due to other causes was still included in the analysis. Third, to test whether effects on primary and secondary outcomes were influenced by plant and time effects, sensitivity analyses were conducted including plant and a categorical time variable as fixed effects.

#### Cost-Benefit

The cost-benefit analysis was performed from the employer’s perspective. Only employer costs and benefits were included. Direct costs were defined as intervention costs, in this case the POSE program, being € 200,- per participant. During this study, no in-company interventions were offered. Benefits were expressed as the difference in monetized outcome measures (absenteeism, presenteeism) between the intervention and control condition. Positive benefits indicate reduced spending. Three metrics were calculated [29]: net benefits (NB = benefits − costs), benefit cost ratio (BCR = benefits/costs), and return on investment (ROI% = (benefits – costs)/costs * 100).

Two analyses were performed on absenteeism costs, one with and another without exceptional causes. Two analyses were performed on absenteeism and presenteeism costs, one with and another without exceptional causes. There were 33 (1.8%) missing values for costs related to absenteeism, caused by the fact that some workers were laid off during the trial. In addition there were 643 (36.0%) missings for costs related to presenteeism, leading to a relatively large number of missings for total costs. Therefore we first performed multiple imputations (n = 20) using chained equations with predictive mean matching. For the analyses on absenteeism costs, the imputation model consisted of the absenteeism costs for every time point, the allocation at every time point, age, day salary and plant. When presenteeism costs were included in the analyses, they were also added to the imputation model. The effect of the trial on the costs was analyzed in these 20 imputed datasets using linear multilevel analyses including a random effect for the individual. Confidence intervals for NB’s, BCR’s and ROI’s were calculated based on 5000 bootstrap samples using the percentile method. Finally, point estimates and lower and upper limits of the confidence intervals were pooled using Rubin’s rules [30]. Cost analyses were performed in STATA 12.1 and 13.1 (StataCorp, College Station, Texas, USA).

## Results

### Non-response Analysis

POSE program participants (n = 303) differed from non-participants (n = 683) on gender, age, job tenure, and sickness absence days per year. Among participants there was a higher percentage of men (89% vs. 78%; p < 0.01), they were older [Med (IQR): 50.6 (9.3) vs. 44.9 (14.9); p < 0.01], worked longer at the company [Med (IQR): 21.8 (17.7) vs. 13.3 (19.2); p < 0.01] and had more sickness absence days [Med (IQR): 2.0 (7.1) vs. 0.0 (5.0); p < 0.01]. Contract hours did not differ (p = 0.52).

### Baseline Characteristics

In total, 305 workers participated in the POSE program. Due to administrative flaws, two participants were excluded from all analyses. Characteristics of the participants are presented in Table 1.

**Table 1:** Personal characteristics at baseline (T_0_), per plant and for the total sample

Outcome	Plant A*	Plant B1*	Plant B2*	Plant E*	Total*
N	110	85	67	41	303
Age, years	49.2 (14.6)	49.5 (10.5)	53.2 (6.7)	51.9 (5.9)	50.6 (9.3)
Gender *n (% male)*	100 (90.9)	79 (92.9)	62 (92.5)	28 (68.3)	269 (88.8)
Job tenure, years	21.9 (11.3)	21.3 (18.9)	24.5 (20.3)	16.4 (14.5)	21.8 (17.7)
Contract hours/4 weeks	144.0 (0.0)	144.0 (0.0)	144.0 (0.0)	152.0 (0.0)	144.0 (0.0)
Sickness absence days/year	2.5 (9.0)	0.0 (7.1)	1.0 (7.1)	2.1 (5.6)	2.0 (7.1)
Education *n (%)*					
No-low	66 (60.0)	56 (65.9)	26 (63.4)	46 (68.7)	194 (64.0)
Medium–high	41 (37.3)	29 (34.1)	14 (34.1)	14 (20.9)	98 (32.3)

*Results are presented as Med (IQR) unless otherwise stated

### Effectiveness Evaluation

#### Missing Data

**Fig. 2:**
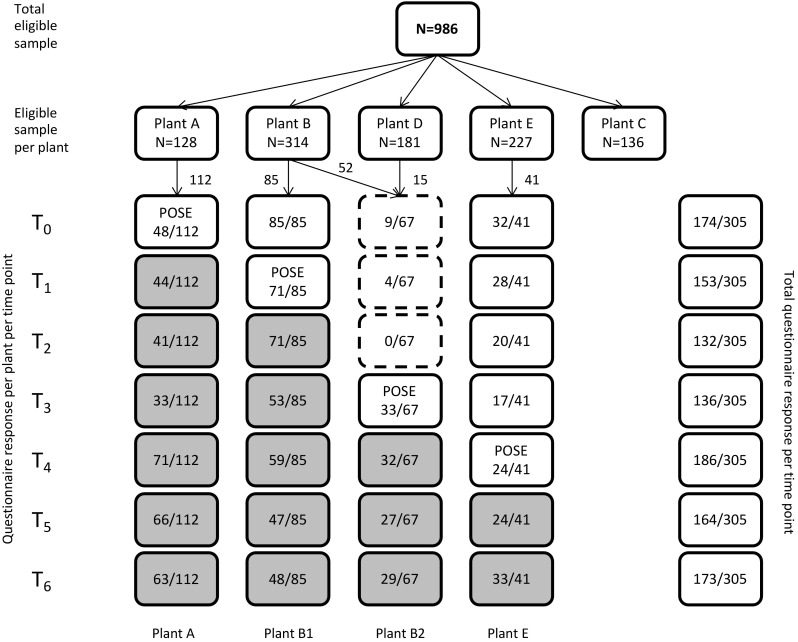
Flow chart of questionnaire response data. *White boxes* refer to periods in the control condition, *grey boxes* refer to periods in the intervention condition. *Dashed lines* refer to plant B2 not yet participating in the study. *Numbers* in the boxes refer to returned questionnaires from the study participants. At the POSE sign, the program was implemented

A flow chart with returned questionnaires per time point is presented in Fig. 2. From a few participants from plant B2, questionnaires were available from the time they worked at plant D (T_0_ and T_1_). Even though questionnaires were returned, individual questionnaire items could be missing. Only complete (sub)scales were included in the analyses. Sickness absence data were complete, as far as people were employed at the time of the study. For the other primary and secondary outcomes, on average 49.3% of the 2,121 items each was missing (N = 303).

#### Descriptive Analysis

**Fig. 3:**
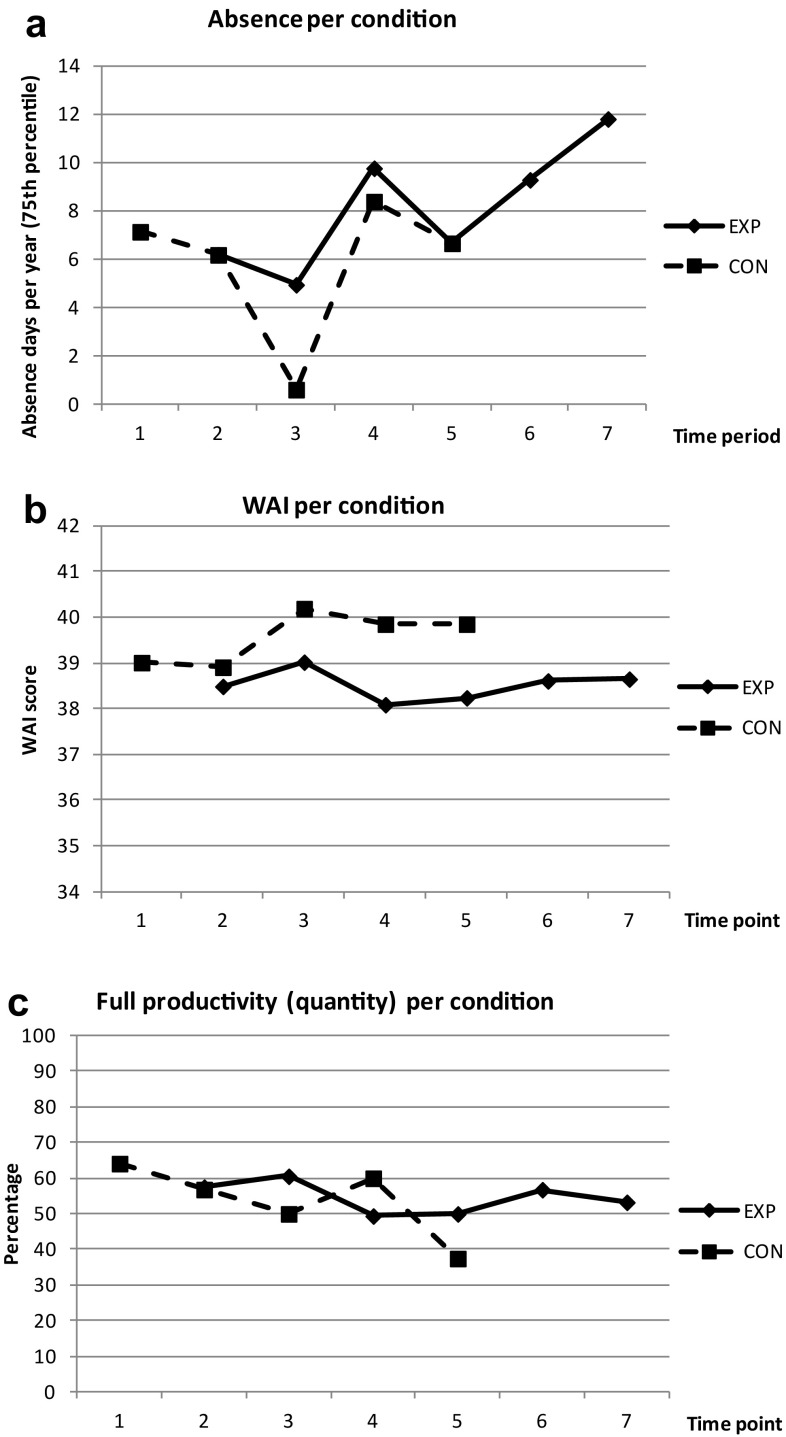
**a** Standardized absence days per year per condition for each time period (75th percentile). **b** Mean work ability scores per condition for each time point. **c** Percentage of participants indicating full quantity of work per condition for each time point. *CON* control condition, *EXP* experimental condition, *WAI* work ability index

Figure 3a–c show the course of absence days, work ability, and productivity respectively throughout the study period.

#### Primary and Secondary Outcomes

Table 2 shows the results of the multilevel analyses for primary and secondary outcome measures (Model 1). Crude models showed statistically significantly negative effects after POSE program implementation for the primary outcome measures. The odds of nine or more absence days was 1.40 (95% CI: 1.09–1.78) and the odds for 100% productivity was 0.71 (95% CI: 0.52–0.96) for the experimental condition compared to the control condition. The mean WAI score was 0.63 (95% CI: 0.14–1.13) points lower for the experimental condition than for the control condition. The effects of Model 1 were virtually unchanged in the analyses adjusted for age (Model 2), except for the effect of the WAI which became smaller and no longer statistically significant. Results from only one analysis of the secondary outcomes showed a significant difference between the control condition and the experimental condition; the psychosocial variable ‘meaning of work’ scored higher in the experimental condition (adjusted analysis: B = 0.18; 95% CI: 0.02–0.35).

**Table 2:** Effects of the POSE program on primary and secondary outcomes

Outcome	Model 1					Model 2				
	OR	B	95% CI		p	OR	B	95% CI		p
*Primary outcomes*										
Sickness absence (all)	**1.40**		**1.09**	**1.78**	**<0.01**	**1.45**		**1.14**	**1.86**	**<0.01**
Sickness absence (excl 200+)	**1.35**		**1.05**	**1.75**	**0.02**	**1.41**		**1.09**	**1.82**	**<0.01**
Sickness absence (excl 100+)	1.26		0.95	1.67	0.12	1.30		0.98	1.73	0.07
Sickness absence (excl exceptional cause)	**1.33**		**1.04**	**1.70**	**0.02**	**1.39**		**1.08**	**1.78**	**0.01**
Work ability		**−0.63**	**−1.13**	**−0.14**	**0.01**		−0.47	−0.97	0.04	0.07
Productivity	**0.71**		**0.52**	**0.96**	**0.03**	**0.70**		**0.52**	**0.96**	**0.03**
Secondary outcomes										
Subjective health		0.01	−0.01	0.02	0.26		0.01	−0.01	0.02	0.27
Health thermometer		−0.39	−1.62	0.83	0.53		−0.39	−1.61	0.84	0.54
Vitality		0.24	−1.47	1.95	0.78		0.23	−1.48	1.94	0.79
Work demands		−0.14	−0.30	0.02	0.09		−0.14	−0.29	0.02	0.09
Work pace		−0.00	−0.18	0.18	0.97		−0.00	−0.18	0.18	0.97
Autonomy		0.07	−0.15	0.29	0.54		0.07	−0.15	0.29	0.55
Possibilities for development		0.13	−0.04	0.29	0.14		0.12	−0.04	0.29	0.15
Meaning of work		**0.18**	**0.02**	**0.35**	**0.03**		**0.18**	**0.02**	**0.35**	**0.03**
Job satisfaction		0.03	−0.02	0.09	0.25		0.03	−0.02	0.09	0.24
Social support from colleagues		0.11	−0.06	0.28	0.21		0.11	−0.06	0.28	0.20
Social support form supervisor		−0.09	−0.28	0.09	0.33		−0.09	−0.28	0.09	0.34
Sense of community		0.09	−0.06	0.25	0.24		0.09	−0.06	0.25	0.24

Model 1 analysis on condition, Model 2 model 1 adjusted for age. All models were adjusted for baseline characteristics. The control condition is used as reference in all analyses. Bold numbers are significant. *95% CI* 95% confidence interval

#### Sensitivity Analyses

After exclusion of participants absent for more than 200 days (n = 17) the difference between conditions was still significant, with the experimental condition having higher odds for nine or more absence days (OR = 1.35; 95% CI: 1.05–1.75). The magnitude of the effect was attenuated and no longer statistically significant when all participants absent for more than 100 days (n = 47) were excluded from the analysis (OR = 1.26; 95% CI: 0.95–1.67). The analysis on sickness absence excluding exceptional causes (n = 16) showed a reduced, but still significant, effect (OR = 1.33; 95% CI: 1.04–1.70). Crude and adjusted models are presented in Table 2.

Two additional models were constructed to control for plant effects and time effects (Appendix). Sensitivity analyses for plant effects (Model 3) did not materially alter the outcomes of primary and secondary outcomes. Sensitivity analyses for time effects (Model 4) showed attenuated and non-significant effect estimates for sickness absence and productivity, but a stronger and significant effect on the WAI score. In addition, the effect on the secondary outcome ‘meaning of work’ disappeared after adjusting for time.

### Cost-Benefit Evaluation

The cost-benefit analysis on absenteeism showed a significant increase in costs after POSE program implementation. Average total benefits per participant were negative and amounted to €-775 (95% CI: €-1077 to €-440). The NB was on average €-975 (95% CI: €-1340 to €-691), indicating that after implementation costs for the employer were higher. The BCR (amount of money returned per Euro invested) was −3.9 (95% CI: −5.7–−2.5) meaning that after implementation of the POSE program costs for the employer were four times higher. The ROI% (percentage of profit per Euro invested) resulted in a loss of 487% (95% CI: −670–−345%).

The additional cost-benefit analysis on absenteeism with exclusion of exceptional causes led to a NB of €-738 (95% CI: €-1057 to €-493), a BCR of −2.7 (95% CI: 4.3–1.5), and a ROI of -369% (95% CI: −528–−247%). The cost-benefit analysis with all absence causes and inclusion of absenteeism and presenteeism costs showed average NB of €-2321 (95% CI: €-2830–€-1836). As shown in the primary analysis, 42% of the NB can be ascribed to absenteeism. BCR amounted to -10.6 (95% CI: −13.1–−8.2), and ROI amounted to −1160% (95% CI: −1415–−918%). The additional cost-benefit analysis on absenteeism and presenteeism with exclusion of exceptional causes led to a NB of €-2040 (95% CI: €-2497–€-1606), a BCR of -9.2 (95% CI: −11.5–−7.0), and a ROI of −1020% (95% CI: −1248–−803%).

## Discussion

After POSE program implementation, no positive effects were found on our primary outcomes of sickness absence days, work ability scores and productivity. With regard to the secondary outcomes, only the average scores on the psychosocial outcome meaning of work were significantly higher in the experimental condition. Other differences were small and non-significant. Financially, after implementation of the POSE program costs were significantly higher than benefits. Based on the present findings, implementation of the POSE program did not improve sustainable employability of workers in the meat processing industry within a 1–3-year follow-up period.

In literature, only few studies were found that also reported on the effect of interventions on sustainable employability. Two studies were performed among older hospital workers (>45 years), one investigating an intervention on problem-solving behavior [31], the other investigating a worksite lifestyle intervention [32]. A third study investigated a worksite prevention program in the construction industry [33]. In these three studies, no or negative effects were reported for sickness absence, work ability, vitality, productivity, and health. A WHS mental module was shown to be effective on work functioning, but the effect was small [12, 34]. A review on WHP programs reported positive effects on sickness absence, work ability, and productivity [35]. Furthermore, similar to our study no effects were found for psychosocial outcomes (work demands, autonomy, and support from supervisor and colleagues), although assessed by different instruments than in the current study [31]. On the other hand, our findings deviated from studies on job-specific WHS programs. A review on job-specific WHS programs showed promising results, although not related to sustainable employability but to physical and mental performance [36].

In the cost-benefit analyses, costs related to absenteeism and self-reported productivity loss were included. Both types of costs are assumed to represent the employer’s perspective. However, in a work environment with little autonomy, such as the meat processing industry, it can be debated whether self-reported productivity should be included in analyses. Workers have to keep up with the pace of conveyor belts. The production process continues even when workers report lower self-reported productivity. This can be explained by the fact that their lower self-reported productivity is compensated by colleagues, or that lower self-reported productivity is poorly related to actual productivity, or a combination of both. Lower self-reported productivity may thus not be a relevant measure from a corporate financial perspective on the short term. However, productivity loss (presenteeism) may be a risk for future health and employability [37, 38]. To enable better interpretation of the results and to add transparency, we have presented the results from the cost-benefit analyses both with and without presenteeism costs.

The results of the cost-benefit analysis of the POSE program deviated from similar studies. A study on sustainable employability in the construction industry showed significantly positive financial benefits, reporting a BCR of 11.0 and ROI of 999% [39]. Although a systematic review on physical activity and nutrition interventions showed negative ROI’s (−112–−49%) these values were much lower than the ROI in the present study [29]. BCR values in that review were marginally negative (−0.12) to somewhat positive (0.51). Another review on workplace wellness programs showed positive results on ROI for absenteeism costs (average of 273%) [40]. No other cost-benefit studies on sustainable employability interventions were identified. Overall, there appears to be insufficient evidence for the financial benefits of sustainable employability interventions.

We cannot rule out that ineffectiveness of the POSE program was caused by program failure [41], because the program was not entirely executed as intended. An extensive process evaluation alongside the present study showed that elements of the POSE program were not properly implemented [42]. The main reason for ineffectiveness may be the poor follow-up of recommendations. Unfortunately, due to lack of registration it was unknown to what extent the recommendations regarding interventions were followed up by the participants and what the effects were. It was known that approximately one out of six participants followed an individual intervention within regular health care after the POSE program, but no company interventions were implemented. Furthermore, but probably of less influence, FCE was not delivered to all participants or not executed according to protocol.

A remarkable finding is that the control condition scored better than the experimental condition on all primary outcomes. Several explanations can be postulated. Firstly, time may have had its influence on effects, since almost all effects became non-significant if controlled for time. Nevertheless, effects remained negative, but decreased in magnitude. For sickness absence, this can be explained by the fact that at the end of the study it markedly increased at all plants, due to diverse causes and irrespective of the time workers had been exposed to the intervention. At that time, all workers were included in the experimental condition due to stepped-wedge design. No workers were in the control condition anymore. A rise in long sickness absence could be postulated as a cause of the increase, although sensitivity analyses showed that long sickness absence could not fully explain this observation. Furthermore, no trend in sickness absence in the control condition was observed, or trends were the same in both conditions. High sickness absence in the last study period may as well have been caused by the flu epidemic in winter of 2014–2015, which was the worst in Dutch history [43]. However, very few workers (5%) exceeded the cut-off value for sickness absence days due to common flu, so this probably did not influence effects to a large extent. Secondly, more sickness absence in the experimental condition could have been caused directly by the POSE program. If workers were at high risk for health loss (e.g., cardiovascular diseases, mental health conditions) this could have resulted in referral to primary care and therefore more sickness absence days in the period shortly after the POSE program. Consequently, this would cause sickness absence to rise in the experimental condition compared to the control condition. From an occupational health care perspective this may be a desired effect, because (short) sickness absence now may prevent longer sickness absence in the future and promote sustainable employability. Thirdly, before implementation of the POSE program the company already invested in reducing sickness absence by both addressing the individual worker and the work environment. Since 2006, sickness absence rates have been reduced from 7 to approximately 4%, which was the baseline absence rate of study participants. This low number may have led to a floor effect and might be an explanation for not finding an additional positive effect for sickness absence. Fourthly, effectiveness of the POSE program can be influenced by company interventions as a result of the program, and is influenced by workers regarding curative and preventive actions. Fifty-five percent of the questionnaire respondents had the intention to act on the recommendations, and 33% indicated to have acted on the recommendations. However, it is unknown to what extent individual workers followed up on recommendations and which specific actions were carried out by the company [42].

Participation in the POSE program was voluntary and partially depended on the invitation strategy, either being automatically enrolled or being invited to subscribe. In plants A and B1 participation was open to all workers, whereas in plants B2 and E only workers aged 50 years and older were invited. When workers were automatically enrolled and had to unsubscribe, participation rates were higher (74–93% vs. 27%). Furthermore, the number of available places in the program was restricted at plants B1, B2, and E. So, not all workers could enter the POSE program during the study. Participants and non-participants differed on age, job tenure, and gender. Differences in age and job tenure can be ascribed to the invitation strategy, which deliberately targeted workers of 50 years and older in plants B2 and E. First inviting these older workers to the POSE program was a strategic choice of the company, because these workers were considered a more vulnerable group [7, 44]. Although sickness absence rates among participants in the present study were at company average, rates were lower in evaluated non-participants. This could lead to the expectation that participants could improve on sickness absence. However, as a result of the sampling strategy our sample was relatively old (median age 50.6 yrs), which in general reduces chances for a positive intervention effect [35]. It was not possible to stratify the results for gender because of the limited number of women (11%) included in the study. However, gender differences did not seem to influence program effectiveness in other studies [35].

### Strengths and Limitations

To our knowledge this study was the first to investigate the effect of a comprehensive WHS program to improve sustainable employability of meat processing workers. The stepped wedge design had the benefit that it was very flexible regarding logistics, i.e. the phased implementation of interventions [45]. In our specific case it allowed the inclusion of a new intervention group, after the drop-out of two plants, although this could have introduced allocation bias, because this inclusion was not done randomly. The design also had the ethical benefit that everyone received the intervention. One of the disadvantages was the complexity of data analysis, since data were available for multiple locations on multiple time points. This made it harder to distinguish possible time effects from plant effects. This was also described in a recently published paper which indicated that the best strategy to analyze stepped wedge designs is not clear yet [46]. Another complexity was the frequency of follow-up measurements which brought along possible respondent fatigue [45]. In our study, participants were followed over a period of three years (except plant B2). Data collection over the long time period has presumably lead to respondent fatigue from T_0_–T_3_, resulting in reduced response rates. This was indicated by respondents as well as by plant managers [42]. From T_4_ onwards response rates were improved by using the strategy to let participants fill out the questionnaires at the workplace, and the provision of a possible incentive (10 x €50,- were raffled) to anyone who completed a questionnaire. Reduced response rates might have led to lower power in the analysis. Fewer participants than required according to the sample size calculation responded to each questionnaire. In plant E too few workers participated in the POSE program, and in plant A and B2 response to the questionnaire was not sufficient at every measurement (e.g., T_3_ and T_4_). This caused almost 50% missing items on primary and secondary outcomes. Nevertheless, missings could be accounted for by the analysis design by introducing a random participant effect, so most probably did not influence effect sizes. Moreover, effects on primary outcomes were significant, indicating sufficient power. A possible limitation may be the approach that was followed in developing the POSE program. The company chose to follow the top-down approach mainly because it was assumed that the workforce consists of low-educated workers with limited knowledge and skills to make appropriate health decisions. However, in the decision process towards implementation of the POSE program, the works council was involved. This party is a representation of workers from all company departments. They were consulted to ensure that the contents of the POSE program did reflect the needs of the workforce in the meat processing industry. Another limitation was that the POSE program was only available for contracted employees. This reduces the available population by 30% which consists of temporary workers. Furthermore, 25% of the workforce is foreign, but most of them are temporary workers. These facts, taken together with no availability of materials in foreign languages, may limit generalizability of study results.

### Conclusions and Recommendations

Primary outcomes did not improve after program implementation and secondary outcomes remained equal after implementation. The program was not cost-beneficial after 1–3 year follow-up. It is important to point out that the POSE program is not a goal in itself, but that it is a strategy to timely intervene and allow restoration of health, improvement of work ability and reduction of sickness absence, i.e. assuring sustainable employability. Regarding detection of workers at risk the program has achieved its purpose, because many workers were identified with one or more risks. A reason for not finding positive effects on primary outcomes may be program failure; in particular, interventions were not deployed as intended. Therefore, future studies should have interventions integrated as part of the workers’ health surveillance program. Interventions should focus both on the individual worker and on the workplace environment. Even though the program may not have a visible positive effect on the short term, it might be beneficial on the long term (5–10 years, or until retirement age). From that perspective, it is recommended to continue the POSE program and evaluate its effectiveness for a longer time period. Furthermore, it is recommended to include a more diverse sample in future studies, including temporary workers and immigrant workers to improve generalizability of the results.

## Appendix

See Table 3.

**Table 3:** Effects of the POSE program on primary and secondary outcomes adjusted for time, plant and interaction effects

Outcome	Model 1					Model 3					Model 4					Model 5				
	OR	B	95% CI		p	OR	B	95% CI		p	OR	B	95% CI		p	OR	B	95% CI		p
Primary outcome																				
Sickness absence (all)	**1.40**		**1.09**	**1.78**	**<0.01**	**1.44**		**1.11**	**1.86**	**<0.01**	1.23		0.84	1.78	0.29	1.12		0.74	1.70	0.60
Sickness absence (excl 200+)	**1.35**		**1.05**	**1.75**	**0.02**	**1.38**		**1.05**	**1.81**	**0.02**	1.23		0.83	1.82	0.31	1.11		0.71	1.73	0.65
Sickness absence (excl 100+)	1.26		0.95	1.67	0.12	1.27		0.94	1.71	0.13	1.16		0.75	1.79	0.51	1.04		0.64	1.70	0.88
Sickness absence (excl exceptional cause)	**1.33**		**1.04**	**1.70**	**0.02**	**1.37**		**1.06**	**1.78**	**0.02**	1.18		0.81	1.73	0.38	1.09		0.71	1.67	0.68
Work ability		**−0.63**	**−1.13**	**−0.14**	**0.01**		−0.49	−1.00	0.02	0.06		**−1.37**	**−2.24**	**−0.5**	**<0.01**		**−1.54**	**−2.54**	**−0.63**	**<0.01**
Productivity	**0.71**		**0.52**	**0.96**	**0.03**	**0.68**		**0.49**	**0.94**	**0.02**	0.89		0.53	1.49	0.66	0.93		0.53	1.62	0.78
Secondary outcomes																				
Subjective health		0.01	−0.01	0.02	0.26		0.01	−0.01	0.02	0.25		0.02	−0.01	0.04	0.24		0.01	−0.01	0.04	0.28
Health thermometer		−0.39	−1.62	0.83	0.53		−0.38	−1 .62	0.86	0.55		0.25	−1.86	2.36	0.82		−0.12	−2.29	2.05	0.91
Vitality		0.24	−1.47	1.95	0.78		0.47	−1.27	2.19	0.60		0.89	−2.04	3.81	0.55		1.07	−1.95	4.09	0.49
Work demands		−0.14	−0.30	0.02	0.09		−0.15	−0.32	0.01	0.06		−0.04	−0.31	0.23	0.80		−0.11	−0.40	0.17	0.44
Work pace		−0.00	−0.18	0.18	0.97		0.01	-0.18	0.19	0.95		0.04	−0.27	0.35	0.80		0.05	−0.26	0.37	0.74
Autonomy		0.07	−0.15	0.29	0.54		0.05	−0.17	0.27	0.67		−0.25	−0.62	0.13	0.19		−0.31	−0.70	0.07	0.11
Possibilities for development		0.13	−0.04	0.29	0.14		0.13	−0.04	0.30	0.15		0.02	−0.27	0.31	0.91		−0.02	−0.32	0.27	0.88
Meaning of work		**0.18**	**0.02**	**0.35**	**0.03**		**0.17**	**0.00**	**0.33**	**0.05**		−0.05	−0.33	0.23	0.72		−0.13	−0.42	0.16	0.38
Job satisfaction		0.03	−0.02	0.09	0.25		0.03	−0.03	0.08	0.34		−0.03	−0.12	0.07	0.57		−0.06	−0.16	0.03	0.20
Social support from colleagues		0.11	−0.06	0.28	0.21		0.14	−0.03	0.31	0.10		−0.15	−0.44	0.14	0.31		−0.12	−0.42	0.17	0.41
Social support form supervisor		−0.09	−0.28	0.09	0.33		−0.08	−0.26	0.11	0.42		−0.16	−0.48	0.16	0.32		−0.18	−0.51	0.14	0.27

Model 1 condition, Model 3 condition adjusted for age and plant, Model 4 condition adjusted for age and time, Model 5 condition adjusted for age, plant and time. All models were adjusted for baseline characteristics. The control condition is used as reference in all analyses
